# HIV-1 Subtype C Unproductively Infects Human Cardiomyocytes *In Vitro* and Induces Apoptosis Mitigated by an Anti-Gp120 Aptamer

**DOI:** 10.1371/journal.pone.0110930

**Published:** 2014-10-17

**Authors:** Walter R. Lopes de Campos, Nthato Chirwa, Grace London, Lia S. Rotherham, Lynn Morris, Bongani M. Mayosi, Makobetsa Khati

**Affiliations:** 1 Emerging Health Technologies Competency Area, Biosciences Unit, Council for Scientific and Industrial Research, Pretoria, South Africa; 2 National Institute for Communicable Diseases, Sandringham, South Africa; 3 Department of Medicine, Groote Schuur Hospital and University of Cape Town, Cape Town, South Africa; Mayo Clinic, United States of America

## Abstract

HIV-associated cardiomyopathy (HIVCM) is of clinical concern in developing countries because of a high HIV-1 prevalence, especially subtype C, and limited access to highly active antiretroviral therapy (HAART). For these reasons, we investigated the direct and indirect effects of HIV-1 subtype C infection of cultured human cardiomyocytes and the mechanisms leading to cardiomyocytes damage; as well as a way to mitigate the damage. We evaluated a novel approach to mitigate HIVCM using a previously reported gp120 binding and HIV-1 neutralizing aptamer called UCLA1. We established a cell-based model of HIVCM by infecting human cardiomyocytes with cell-free HIV-1 or co-culturing human cardiomyocytes with HIV-infected monocyte derived macrophages (MDM). We discovered that HIV-1 subtype C unproductively (i.e. its life cycle is arrested after reverse transcription) infects cardiomyocytes. Furthermore, we found that HIV-1 initiates apoptosis of cardiomyocytes through caspase-9 activation, preferentially via the intrinsic or mitochondrial initiated pathway. CXCR4 receptor-using viruses were stronger inducers of apoptosis than CCR5 utilizing variants. Importantly, we discovered that HIV-1 induced apoptosis of cardiomyocytes was mitigated by UCLA1. However, UCLA1 had no protective effective on cardiomyocytes when apoptosis was triggered by HIV-infected MDM. When HIV-1 was treated with UCLA1 prior to infection of MDM, it failed to induce apoptosis of cardiomyocytes. These data suggest that HIV-1 causes a mitochondrial initiated apoptotic cascade, which signal through caspase-9, whereas HIV-1 infected MDM causes apoptosis predominantly via the death-receptor pathway, mediated by caspase-8. Furthermore the data suggest that UCLA1 protects cardiomyocytes from caspase-mediated apoptosis, directly by binding to HIV-1 and indirectly by preventing infection of MDM.

## Introduction

South Africa has the highest HIV-1 prevalence that is almost exclusively subtype C [4]. Chronic HIV-1 infection causes a broad range of clinical complications, some of which remain poorly elucidated. One such disease is HIV-associated cardiomyopathy (HIVCM). HIVCM is characterized by the loss of cardiomyocytes and their replacement by fibrous tissue due to chronic HIV-1 infection. The pathogenesis of HIVCM remains incompletely understood, although direct and indirect effects of HIV infection are thought to play a role [1], [2], [3].

There is increasing clinical and experimental data that point to a direct role of HIV-1 and its associated proteins as important causes of HIVCM [4], [5], [6], [7], [8]. HIV-1 infection of human cardiomyocytes has been observed in cardiac biopsies of HIV positive patients [9], [10], [11], although its role in the pathogenesis of the disease is controversial. More clear is the role played by its structural, surface envelope glycoprotein called gp120, which has been reported to cause apoptosis of rodent cardiomyocytes [4], [5], [6], [7], [8]. The cardio toxic effects are believed to be triggered in a caspase-dependent, mitochondria-initiated fashion, when gp120 interacts with the CXCR4 receptor on the surface of cardiomyocytes [12]. This in turn results in the induction of a negative ionotropic effect through increase in nitric oxide (NO) production via p38 mitogen activated protein kinase (p38 MAPK)-iPLA2-troponin I initiated NF-κB activation [6], [7], [8]. The end result of NO production is free-radical insult through the formation of reactive oxygen species (ROS), leading to the loss of mitochondrial membrane potential and membrane permeabilization, in a Bcl-2-inhibitable manner [13], [14]. The exact pathway is not fully understood but leakage of cytochrome *c* into the cell cytosol following permeabilization of the mitochondrial membrane triggers the activation of the caspase 9/3 complex eventually culminating in nuclear DNA fragmentation [15].

The apoptotic signal is not restricted to host cell interaction with viral proteins. Cytokines secreted by HIV-infected cells, primarily infiltrating macrophages, also play a role in the development of HIVCM. Tumor necrosis factor (TNF) has been suggested to be the main cause in murine cells [16], [17]. It interacts with its respective death receptor, TNF-R1, leading to extrinsic or death-receptor mediated apoptosis [16], [17]. However, *in vitro* apoptosis triggered by gp120 is initiated preferentially through the CXCR4 receptor than the death-receptor. Analysis of cardiac biopsies revealed strong expression of both caspase 9 and TNF, which was detected in infiltrating inflammatory cells of HIVCM patients, but not in HIV-infected patients without HICVM, making a strong argument towards death-receptor initiated apoptosis *in vivo* [4]. These suggest that both pathways are at play, but why and under which conditions each pathway is favored remains unclear.

In this paper, we report the HIV-1 infection kinetics of well characterized human cord-blood stem cell-derived cardiomyocytes as well as the associated apoptotic pathways and receptors involved during HIV-cardiomyocyte interaction. Importantly, we also show that a shortened and synthetic derivative of gp120 binding and HIV-1 broadly neutralizing aptamer called UCLA1 [18] mitigated HIV-initiated apoptosis in the cardiomyocytes.

## Results

### Phenotyping human cardiomyocytes

**Figure 1:**
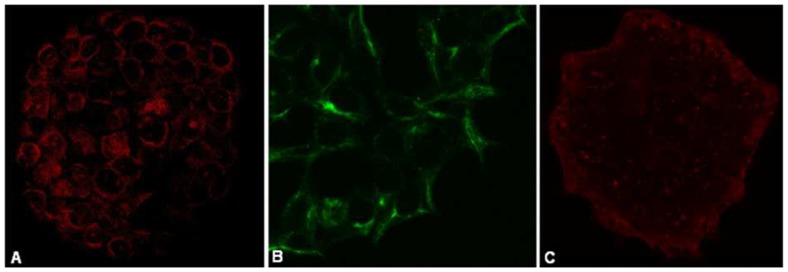
Phenotyping cultured human cardiomyocytes. Human cardiomyocytes cultured on microscope cover slips where phenotyped using cardiac markers (A) α-MHC (63× magnification) (AF546-labeled secondary antibody [red]) (B) Cx43 (x40) (AF488-labeled primary antibody [green]) and (C) cTnT (40× magnification) (AF546-labeled secondary antibody [red]). Cardiomyocytes where imaged on a Zeiss 780 immunofluorescent confocal microscope.

To confirm the phenotype of the human cord-blood stem cell-derived cardiomyocytes, we stained the cells with antibodies for known cardiac cell markers. The cells stained positive for canonical cardiac specific markers such as α-MHC, Cx43 and cTnT, respectively (Figure 1A–C).

### HIV-1 entry into cardiomyocytes is co-receptor independent and abortive

**Figure 2:**
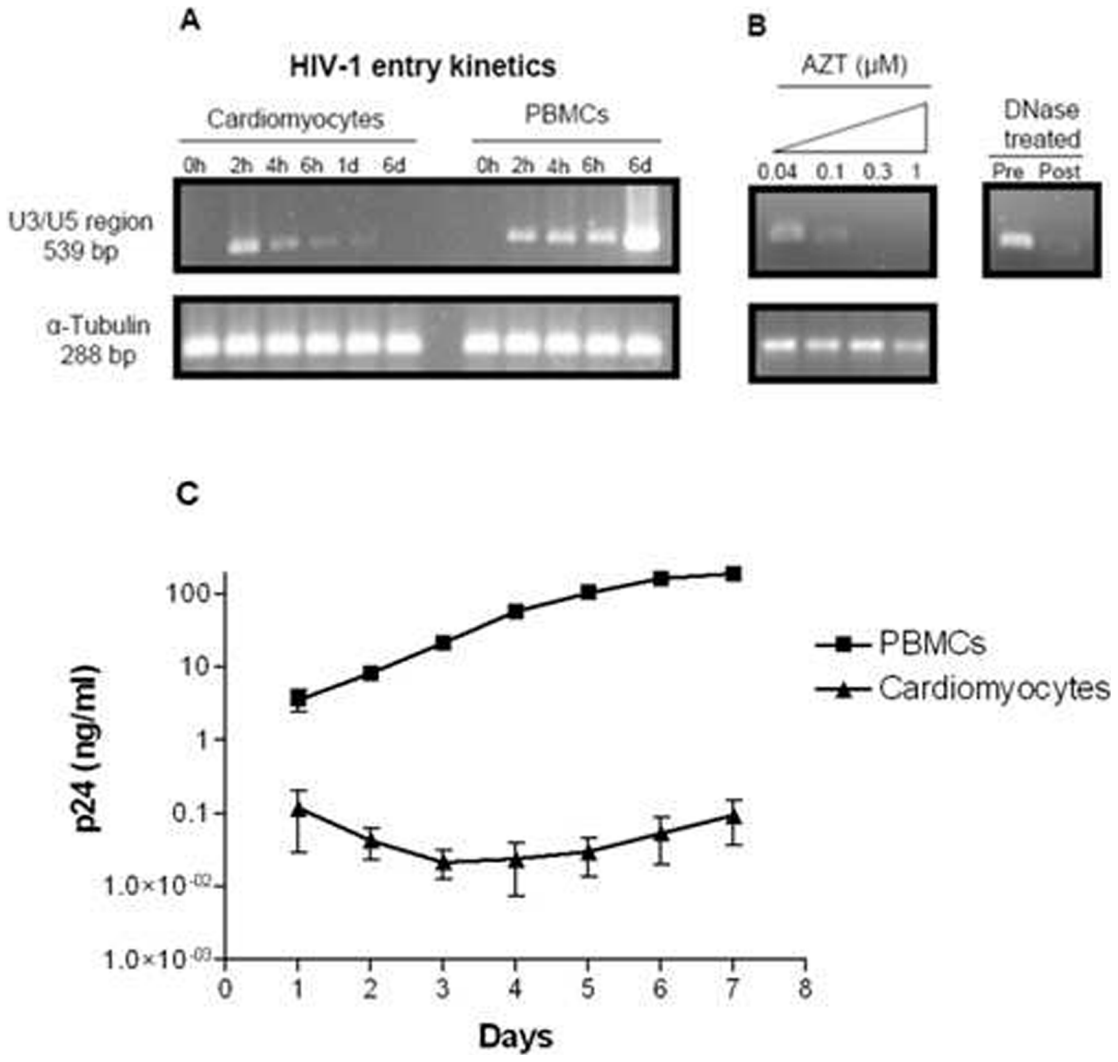
Kinetics of HIV-1 infection of CM and PBMC. (A) Agarose-gel electrophoresis of the PCR-amplified U3/U5 region of a dual tropic (R5X4) HIV-1 subtype C isolate called HIV-1_CM9_ infected culture lysates at different times after infection. The 539 bp fragment indicates the presence of proviral DNA. A 288 bp region of the gene coding for α-tubulin is a control ensuring equal loading of the wells. (B) Proviral DNA in CM exposed to serial dilutions of AZT. (Insert) Agarose-gel electrophoresis of cell-free HIV-1_CM9_ culture digested with DNaseI for 1 h prior to infection. (C) Detection of replication of HIV-1_CM9_ in CM and PBMC using p24 ELISA.

To establish the cell-based HIVCM model and investigate the effect of HIV-1 on human cardiomyocytes, we first evaluated if different HIV-1 subtype C strains, as defined by co-receptor usage, can productively infect human cardiomyocytes. Cultured human cardiomyocytes were infected by all the R5, X4 and R5/X4 subtype C HIV-1 isolates tested in the presence or absence of respective co-receptor inhibitors as evidenced by the presence of proviral DNA (Table 1). While all the tested subtype C HIV-1 isolates also infected PBMC, we observed successful inhibition of infection of PBMC in the presence of specific inhibitors of the relevant co-receptors (Table 1). In HIV-1_CM9_ infected cardiomyocytes, we observed time-dependent peak in pro-viral DNA 2 hours post infection and subsequent decrease to almost undetectable levels after 24 hours and complete absence after 6 days (Figure 2A). This contrasted with the infection kinetics observed in PBMC in which there was a time-associated increase of pro-viral DNA (Figure 2A). Synthesis of pro-viral DNA in cardiomyocytes was inhibited by zidovudine (AZT) in a dose-dependent manner (Figure 2B). The absence of p24 viral capsid protein in cardiomyocytes, measured daily over 7 days, suggested that HIV-1 cannot replicate inside cardiomyocytes; as opposed to replication in PHA-treated, IL-2 stimulated primary PBMC as measured by an increase in p24 viral protein (Figure 2C). Taken together, the data suggest that while HIV-1 infects cardiomyocytes, the virus does not enter using the classic co-receptors and the infection is non-productive.

**Table 1:** HIV-1 infection of cultured human cardiomyocytes (CM) and peripheral blood mononuclear cells (PBMCs), activated with phytohemagglutinin (PHA) and treated with interleukin-2 (IL-2)) in the presence of inhibitors.

		Entry Inhibitors							
		No inhibitors		AMD3100 (500 nM)		RANTES (100 nM)		UCLA1 (100 nM)	
HIV Strain	Tropism	CM	PBMC	CM	PBMC	CM	PBMC	CM	PBMC
SW2	R 5	+	+	+	+	+	−	+	−
SW4	R 5	+	+	+	+	+	−	+	−
									
SW12	X4	+	+	+	−	+	+	+	−
TM46b	X4	+	+	+	−	+	+	+	−
									
CM9	R5X4	+	+	+	−	+	+	+	−
DU179-99	R5X4	+	+	+	+	+	+	+	−
RP1	R5X4	+	+	+	+	+	+	+	−

The presence of pro-viral DNA as indicated by the presence of a 539 bp fragment amplified from the U3/U5 region was indicative of viral entry and is shown as +. PCR negative samples were considered entry negative and are shown as −. This study was done in triplicates and repeated twice.

### Apoptosis of cardiomyocytes is predominantly triggered by X4 viruses and is down regulated by AMD3100 and UCLA1

**Figure 3:**
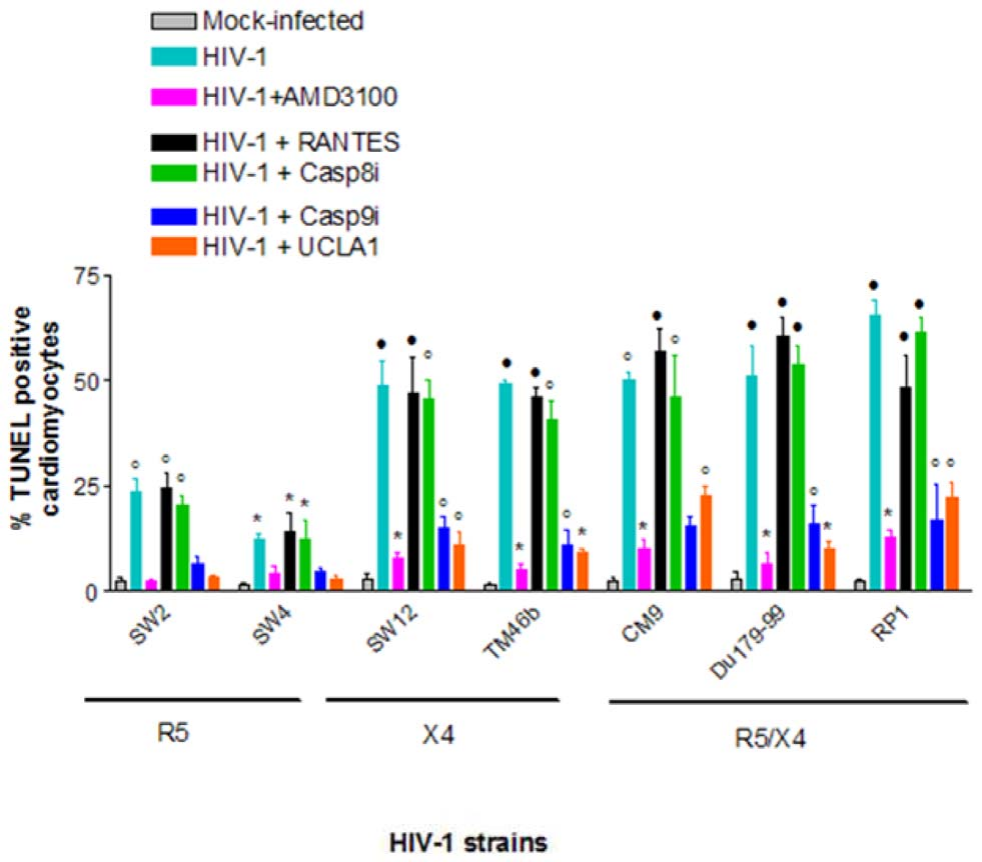
Quantification of the relationship between HIV-1 tropism and induction of CM apoptosis using TUNEL assay. Statistically significant TUNEL positive results compared to mock-infected control cells were determined by the student *t*-test and are marked as: *, *P*<0.05; ·, *P*<0.005; °, *P*<0.0005 (n = 3± SEM).

After establishing that all R5, X4 and R5X4 HIV-1 subtype C isolates tested unproductively infected cardiomyocytes, we next investigated whether or not they induce apoptosis of cardiomyocytes to further probe HIV-cardiomyocyte interaction in our cellular model. Induction of apoptosis in cardiomyocytes by X4 viruses was on average 3 times higher than that of R5 viruses– 54%±6.6% TUNEL positive for X4 viruses vs. 18%±5.3% for R5 viruses (Figure 3). Dual-tropic (R5X4) viruses also induced considerably more apoptosis in cardiomyocytes than R5-tropic viruses; by a magnitude of 2.7 (Figure 3). When R5 viruses were pre-incubated with UCLA1 before infecting cardiomyocytes, apoptosis was reduced from an average of 18%±5.3% to background or mock infected levels (Figure 3). AMD3100 also reduced apoptosis of R5 infected cardiomyocytes from 18%±5.3% to about mock infected levels (Figure 3). Reduction in apoptosis was stronger in cardiomyocytes infected with X4 and R5/X4 viruses, respectively, where on average the TUNEL positive population decreased from 52%±6% to 5%±3% (*P*<0.001) in the presence of AMD3100 (Figure 2). The UCLA1 aptamer did not show a strong protective effect as AMD3100 but the percentage of TUNEL positive cardiomyocyte decreased from 52%±6% to 13%±6% (*P*<0.001) when compared to untreated but HIV-1 infected cardiomyocyte (Figure 3). On the other hand, RANTES, the physiological ligand for the CCR5 chemokine receptor, had no statistically significant effect in protecting cardiomyocytes from HIV-1 associated apoptosis when compared to untreated but HIV-1 infected cardiomyocytes (Figure 3). Collectively, these data suggest that apoptosis of cardiomyocytes is triggered through the CXCR4 receptor, predominantly by X4 viruses and is down regulated by AMD3100 and UCLA1.

### HIV-1 initiates apoptosis of cardiomyocytes preferentially through the intrinsic or mitochondrion-initiated pathway mediated by caspase 9 activation

**Figure 4:**
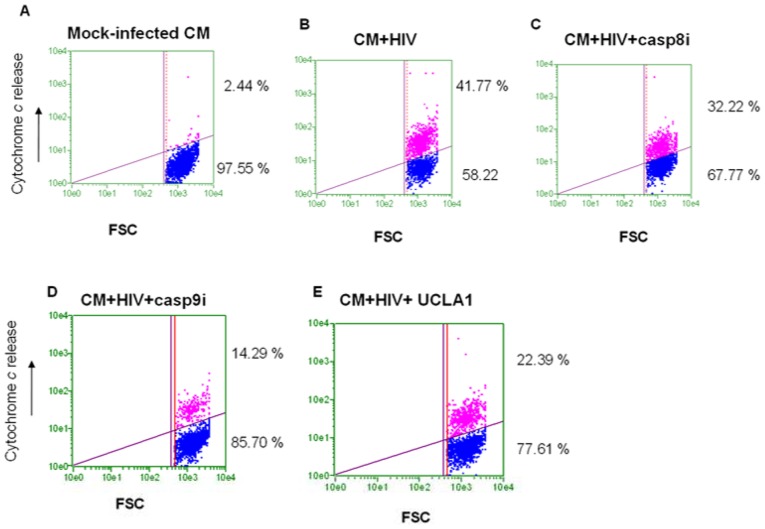
Cytochrome *c* release in HIV-1_CM9_-infected CM. CM were either (A) mock infected; (B) HIV-1 infected; (C) infected in the presence of casp8i; (D) infected in the presence of casp9i and (E) infected with virus pre-incubated with UCLA1. Data were presented in dot plots relating fluorescent intensity as a measure of cytochrome *c* release against forward scatter.

Cytochrome *c* leakage from the mitochondria as well as caspase 9 activation are markers of the intrinsic mitochondrial-initiated pathway and caspase 8 activation is the canonical caspase of the extrinsic apoptotic pathway. In this experiment we investigated the preferred apoptotic pathways in cardiomyocytes directly infected by HIV-1. We found that in the untreated control cardiomyocytes, only 2.4%±0.7% cells stained positive for cytochrome *c* release (Figure 4A). On the other hand, 41.77%±3.0% of HIV-infected cardiomyocytes stained positive for cytochrome c release (Figure 4B). The preferential caspase 8 inhibitor reduced cytochrome c release in HIV-1 infected cardiomyocytes to 32.2%±5.3% (Figure 4C). The preferential caspase 9 inhibitor significantly reduced cytochrome c release in HIV-1 infected cardiomyocytes to 14.3%±3.6% (Figure 4D). UCLA1 also significantly reduced cytochrome c release in HIV-1 infected cardiomyocytes to 22.4%±4.4% (Figure 4E).

**Figure 5:**
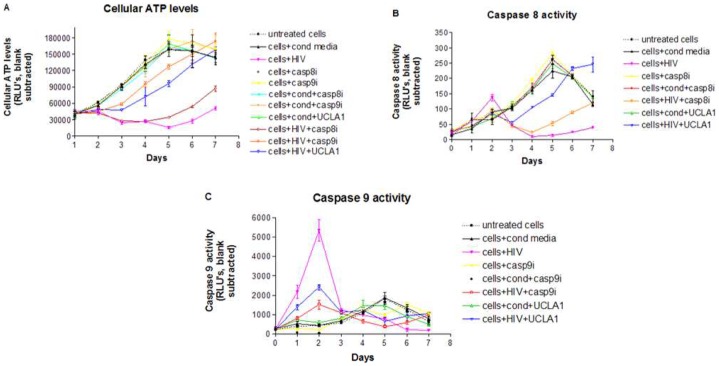
Caspase 8 and 9 activities in CM infected with HIV-1. CM were exposed to: media alone; conditioned media containing virus; HIV; 100 µM of casp8i; 100 µM of casp9i; conditioned media and 100 µM of casp8i; conditioned media and 100 µM of casp9i; conditioned media and 100 nM UCLA1 aptamer; HIV and 100 µM of casp8i; HIV and 100 µM of casp9i; HIV pre-incubated for 1 h with 100 nM of UCLA1 aptamer. Cells were harvested daily for 7 days. (A) ATP levels were measured as a correlate of cell viability; (B) caspase 8 and (C) caspase 9 activities were measured as indicators of extrinsic and intrinsic apoptosis, respectively. All assays were luminescence-based and presented as graphs relating RLU to number of days (n = 3± SEM).

There was an inversely proportional link between total cellular ATP, which was used as marker of enhanced oxidative stress and hence a surrogate measure of cellular viability, and caspase 9 activation during HIV-1 infection of cardiomyocytes (Figure 5). A drop in ATP levels was observed 1 day after peak caspase 9 activity and subsequent recovery at day 5 correlated with a decrease in caspase 9 levels. Both caspase 9i and UCLA1 protected HIV-1 infected cardiomyocytes from cell death as indicated by an exponential increase in cellular ATP from day 3 to 7 (Figure 5A).

Caspase 8 activation in HIV-1 infected cardiomyocytes was not significantly higher from the untreated control cells or any other test condition (Figure 5B). Furthermore, there was no statistical difference (*P* = 0.5) between cells treated with caspase 8i or UCLA1 and untreated, infected cells up to day 3 (Figure 5B). There was however an exponential increase in enzyme activity levels in infected, UCLA1 treated cells, very similar to untreated controls from day 3 to 7 (Figure 5B). The opposite was observed with caspase 8i treated cells, where there was a decline in enzyme activity up to day 4 followed by an increase up to day 7 (Figure 5B).

Peak caspase 9 enzyme activity was observed two days post-exposure to HIV-1, resulting in a 9.3 fold increase in enzyme levels, when compared to the untreated controls (Figure 5C). This was followed by a sharp decrease in enzyme activity relative to control cells. The preferential caspase 9 inhibitor significantly reduced the enzyme activity in the presence of HIV-1 at days 1–2 of treatment (Figure 5C). UCLA1 also had a modulatory effect in caspase 9 activity during HIV-infection infection (RLU  = 2410, *P* = 0.001), albeit weaker than that of casp9i (RLU  = 1515, *P* = 0.02). All other test conditions did not significantly affect caspase 9 levels (*P*>0.07) during the entire duration of the experiment (Figure 5C). Taken together, the data suggest that both intrinsic and extrinsic apoptosis are perhaps triggered by HIV-1 in cardiomyocytes, however the intrinsic or mitochondrion initiated pathway is probably dominant as indicated by cytochrome c release and the high levels of caspase 9 activity in HIV-1 infected cardiomyocytes. Thus caspase 9 is possibly a key player in apoptosis of HIV-1 infected cardiomyocytes since its inhibition resulted in significant reduction of cytochrome c release and almost completes abrogation of apoptosis in HIV-1 infected cardiomyocytes, while inhibition of caspase 8 had a much less pronounced effect.

### HIV-1 infected macrophages preferentially trigger extrinsic or death receptor apoptotic pathway in cardiomyocytes

**Figure 6:**
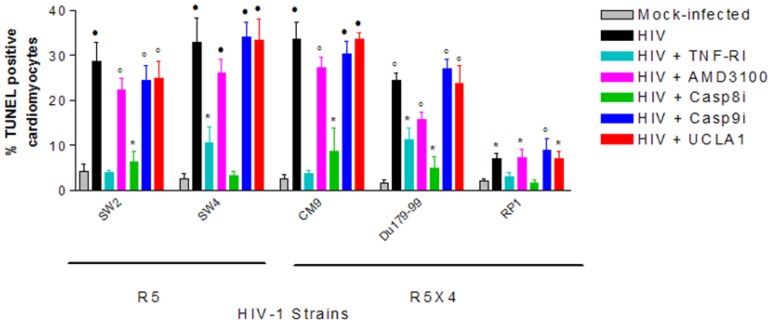
CM apoptosis in a co-culture model with HIV-infected MDM. MDM were infected with the R5-tropic viruses SW2, SW4 or the dual tropic viruses CM9, DU179-99 and RP1 for 24 h. Uninfected CM were added and the co-cultures were then incubated for a further 24 h. Following incubation, the cell monolayers were stained for TUNEL detection. Statistically significant TUNEL positive results compared to mock-infected control cells were determined by the student *t*-test and are marked as: *, *P*<0.05; ·, *P*<0.005; °, *P*<0.0005 (n = 3± SEM).

There is evidence suggesting that infiltrating HIV-1 infected macrophages in cardiac tissue are strongly associated with caspase-dependent cardiomyocyte apoptosis. Therefore, to mimic the *in vivo* environment and to help further establish the HIVCM cellular model, we investigated if MDM experimentally infected with HIV-1 can induce apoptosis of cardiomyocytes-MDM co-cultures. MDM infected with R5 and R5X4 viruses significantly induced apoptosis of cardiomyocytes-MDM co-cultures compared to mock-infected MDM (Figure 6). AMD3100 (*P*>0.09) and UCLA1 (*P*>0.07) did not significantly protect cardiomyocytes from apoptosis induced by HIV-1 infected MDM (Figure 6). However, significant protection against apoptosis was achieved in the presence of TNF-R1 (*P*<0.02) and casp8i (*P*<0.02), which are both key components of the extrinsic apoptotic pathway (Figure 6). Thus, the data suggest that HIV-1 infected MDM induce apoptosis in uninfected cardiomyocytes-MDM co-cultures via the extrinsic or death receptor pathway.

## Discussion

In this study, we have showed for the first time that UCLA1 [18], a synthetic derivative of gp120 binding aptamer called B40 [19], [20], [21], [22], mitigated HIV-1 subtype C induced apoptosis of human cord-blood stem cell-derived cardiomyocytes. We showed that these stem cell-derived human cardiomyocytes expressed Tropic T, Connexin 43 and α-MHC cardiac specific canonical markers in addition to the Troponin 1 marker, which we showed that they also express in our recent publication [21]. Thus, these data extend earlier findings that these cells expressed Nkx2.5, GATA-4, MEF2C, SERCA2a, α-MHC, α-sarcomeric actin [23] and Troponin I [21], [23]. In addition to being positive for the immunological cardiomyocytes markers, these cells were shown to be capable of synchronized contraction [23]. These human cord-blood stem cell-derived cardiomyocytes used in this study, are similar to recently reported human embryonic stem cell-derived cardiomyocytes, which are also capable of spontaneous beating [24].

Furthermore, we also showed that while HIV-1 subtype C infects the human cardiomyocytes, it does not necessarily enter using the classic CCR5 or CXCR4 co-receptors. These data indirectly confirm and extend previous studies using rat heart cells, where it was shown that HIV-1 subtype B entered cultured neonatal rat ventricular myocytes by macropinocytosis but did not replicate [4], [5]. We also observed that despite entering human cardiomyocytes, a dual tropic (R5X4) HIV-1 subtype C isolate called HIV-1_CM9_ was unable to complete its life cycle, as indicated by the time-dependent decrease in pro-viral DNA upon infection as well as by the absence of p24 antigen in culture supernatants of HIV-1 infected human cardiomyocytes, further extending earlier findings by others [4], [5]. It is likely that the pro-viral pre-integration, nuclear-localization complex is never formed resulting in a time-dependent degradation of the pro-viral DNA in the cytoplasm. In addition to HIV-1 previously shown to infect rat ventricular myocytes [4], [5], there is also *in vivo* clinical evidence suggesting direct infection of cardiomyocyte in AIDS patients [10], [11] but these studies were limited to detecting viral nucleic acid and proteins in cardiac biopsies, which is a mixture of different cells including macrophages. Thus, our study does not only confirm and extend earlier findings by others [4], [5] but it is the first to investigate whether or not HIV-1 in general and subtype C in particular, could directly infect cultured human cardiomyocytes. Our study is also the first to show that monocyte derived macrophages infected with HIV-1 could cause apoptosis of cultured human cardiomyocytes.

Our data have also revealed that although HIV-1 subtype C did not complete its life cycle in human cardiomyocytes, it was still capable of inducing significant caspase-dependent apoptosis. Furthermore, entry was not required for apoptosis since the apoptotic signal is probably initiated by interaction with the cell-surface receptors, the CXCR4 chemokine receptor and the TNF-R1 death receptor. The predominant apoptotic pathway varied from CXCR4-triggered, mitochondrion-initiated to CD95/Fas-ligand initiated, depending on the culture conditions as previously reported in T cells [25], [26]. In the absence of monocyte derived macrophages, cell free HIV-1 possibly interacts directly with the CXCR4 chemokine receptor on cardiomyocytes [12], resulting in cell death. This death-inducing signaling however was significantly stronger using X4 and dual tropic (R5X4) viruses, possibly because these viruses interact with higher affinity with the CXCR4 receptor than R5 tropic viruses. The UCLA1 binding site maps to the co-receptor binding region of gp120 on HIV-1 [18], [20], [22], [27], which may explain its strong anti-apoptotic properties, very similar to that brought about by the highly selective CXCR4 receptor ligand AMD3100. This observation, together with cytochrome *c* leakage into the cell cytosol points to a CXCR4/HIV-1 interaction as the key apoptosis trigger as recently reported in murine cardiomyocytes [12] and earlier in primary human macrophages [28], resulting in mitochondrial initiated apoptosis [4], [25] independent of death-receptor mediated signaling [29]. It has been reported that the CXCR4 receptor is involved in both caspase-dependent and caspase-independent apoptosis induction [12]. There is however some controversy regarding the circumstances under which each pathway is favored. Some groups have reported that caspase-dependent apoptosis requires interaction with both CD4 and CXCR4 receptors, and in the absence of CD4, the apoptotic signal initiated by CXCR4 is caspase-independent [26], [30], [31]. On the other hand, another study conducted by Roggero and co-workers revealed that CXCR4 cooperation with a CD4 signal was not required for the induction of mitochondrial-mediated apoptosis [25]. Furthermore, it was previously shown that deletion of the cytoplasmic tail of CD4 in cells co-transfected with both CD4 and CXCR4 did not shift the apopotic pathway from caspase-dependent to caspase-independent [32], [33].

In this study, we also observed that both apoptotic pathways were intimately related with caspase activity, an observation that is not supported by others who used gp120 and T-cells [31]. This discrepancy could be explained by the fact that we used fully infectious HIV-1 subtype C primary isolates with trimeric envelope and macrophage-cardiomyocyte co-cultures instead of recombinant monomeric gp120 and T-cells. Notwithstanding, the 9-fold increase in cellular caspase 9 levels, and the improved cell survival brought about by its inhibition, further strengthens the argument for a caspase-dependent apoptotic pathway.

In addition, we observed a second, caspase-dependent apoptotic pathway, initiated at the TNF-R1 death receptor in the presence of HIV-1. Although the peak in caspase 8 activity was not statistically significant, there was a significant degree of cellular protection when HIV-infected cardiomyocytes were treated with the preferential caspase 8 inhibitor Z-IETD-FMK. Caspase 8 is regarded as a key initiator of death receptor mediated apoptosis and under physiological conditions, it is primed by the TNF superfamily member of related apoptosis triggers [34], [35]. Our case for extrinsic apoptosis rests on the merit of its inhibition rather than on up-regulation of its downstream effector caspase.

The macrophage-cardiomyocyte co-culture model shows a drastic shift in the preferential apoptotic trigger, towards the extrinsic pathway, which is likely initiated by interaction with TNF-R1 secreted by death-signaling molecules produced by HIV-1 infected monocyte derived macrophages. Under these conditions apoptosis progressed with much more rapid kinetics, measured in hours as opposed to days when initiated by the CXCR4 receptor. The almost complete abrogation of apoptosis in the presence of TNF-R1 as well as casp8i sheds further light into preferential apoptotic pathways and receptor trigger during HIV-1 infection of cardiomyocyte. In this scenario, the UCLA1 had no impact in apoptosis modulation, which was expected since the extrinsic or death-receptor initiated apoptosis was most likely triggered by monocyte derived macrophages, rather than viral factors. Nonetheless, by preventing macrophage infection, UCLA1 can strongly modulate both apoptotic pathways. A further advantage of UCLA1 over conventional anti-retrovirals is that it probably neutralizes the deleterious effects of cell-free virus by binding to gp120 envelope protein and preventing it from interacting with the CXCR4 chemokine receptor and thereby preventing or reducing apoptosis. This reasoning is consistent with the observation that protease inhibitors used in anti-retroviral therapy give rise to non-infectious virions with fully functional envelope glycoproteins that have been observed to lead to apoptosis of non-infected CD4^+^ and CD8^+^ T-cells [36]. Since the ratio of circulating non-infectious to infectious virions is exponentially greater [37], [38], the role played by these non-infectious virus particles in the progression to cardiovascular disease cannot be ignored, particularly in cell expressing the CXCR4 chemokine receptor. Therefore, it is perhaps erroneous to assume that because these viral particles are not capable of productive infection, that they play no role in HIV-1 pathogenesis, which includes HIV-associated cardiomyopathy.

In summary, this study demonstrated that HIV-1 subtype C unproductively infects human cord-blood stem cell-derived cardiomyocytes and, while the virus did not replicate in the human cardiomyocytes, it directly (cell free virus) and indirectly (macrophage-associated virus) induced apoptosis, which could be mitigated by UCLA1 in culture. The limitation of this study is the use of human cord-blood stem cell-derived cardiomyocytes, which formed clumps in culture and hence seemed not to exhibit a distinct morphology and antibody staining pattern usually observed in primary cardiomyocytes. The future research challenge will be to extend this proof-in-principle observation that UCLA1 can protect human cardiomyocytes from both direct and indirect effects of HIV-1 in human heart tissue samples and in an advanced model of HIV-1 associated cardiomyopathy.

## Materials and Methods

### Ethics statement

The human cord-blood stem cell-derived cardiomyocytes were purchased from Celprogen Inc. (USA) and human buffy coats, which are the residual material after plasma, platelets, and red cell separation for patient use, were supplied by the South African National Blood Services. The investigation with human cells conformed to the principles outlined in the Declaration of Helsinki [39] and approval was granted by the human research ethics committee of the University of Witwatersrand (protocol number M071031).

### Cell cultures

The human cord-blood stem cell-derived cardiomyocytes were cultured as previously described [21], [23].

Peripheral-blood mononuclear cells (PBMC) and monocyte derived macrophages (MDM) were isolated from heparinized human buffy coats of normal, HIV-negative donors and cultured as previously described [27].

### Phenotyping the cardiomyocyte

To confirm the cardiac phenotype of the human cord-blood stem cell-derived cardiomyocytes, we stained the cells with antibodies for known cardiac specific markers. Cardiomyocytes were seeded on cover slips obtained from Celprogen (Celprogen Inc, USA) and fixed with ice-cold methanol for 30 min. Primary antibodies diluted 1∶100 with blocking buffer (PBS containing 1% bovine serum albumin (BSA) were applied on cover slips for 1 h at room temperature. Primary monoclonal antibodies included mouse anti-alpha myosin heavy chain (α-MHC) (Abcam plc, UK), mouse anti-connexin43 (Cx43) Alexa Fluor 488 conjugated (Invitrogen, USA), and goat polyclonal anti-Troponin T (cTnT) (Santa Cruz Biotechnology, USA). Cardiomyocytes were washed four times with ice-cold PBS, then incubated with 1∶200 dilution of labelled secondary partner antibodies, which included donkey anti-goat Alexa Fluor 546 conjugate (Invitrogen, USA) and goat anti-mouse Alexa Fluor 546 conjugate (Invitrogen, USA). The cardiomyocyte monolayer was covered with a standard glass slide in the presence of mounting medium containing 4′-6-Diamidino-2-phenylindole (DAPI) nuclear stain (Santa Cruz Biotechnology, USA) and imaged using an immunofluorescent confocal microscope (Zeiss LSM780, Germany).

### HIV-1 isolates and virus conditioned media

A panel of 7 previously reported [40], [41] clinical isolates of HIV-1 subtype C were used. This included two R5 viruses (SW2, SW4), two X4 viruses (SW12 and TM46b) and three R5X4 viruses (CM9, Du179-99 and RP1).

Virus conditioned media was prepared from day 5 culture supernatants of HIV-infected phytohemagglutinin (PHA)-stimulated IL-2 treated PBMC. Virus was separated from the supernatants by size-exclusion centrifugation using a 200 KDa NWM filter (Millipore, USA). This allowed for the separation of cell-free virions from cellular debris and viral proteins.

### Anti-HIV-1 molecules and aptamer

Zidovudine (AZT), AMD3100 and RANTES (regulated upon activation, normal T-cell expressed, and secreted) were obtained through the AIDS Research and Reference Reagent Program, Division of AIDS, NIAID, NIH, USA. A fully characterized UCLA1 aptamer, previously reported [18] was donated by William James, University of Oxford, United Kingdom. Briefly, UCLA1 is a shortened and synthetic derivative of gp120 binding and HIV-1 neutralizing aptamer called B40 [19], [20], [21], [22].

### Infection of cells with HIV-1 and PCR detection of proviral DNA

A total of 5×10^4^ cardiomyocytes or PBMC cultured in 96-well plates (Corning, USA) were infected with HIV-1 at a concentration of 200 ng/ml p24 in the absence or presence of 500 nM of the entry inhibitor AMD3100, 100 nM RANTES or 100 nM of UCLA1 aptamer. Following incubation for different time intervals, protein digestion and inactivation of proteinase K (10 min at 95°C) the cell lysate was subjected to PCR amplification as previously described [42].

### Detection and modulation of HIV-1 initiated apoptosis in cardiomyocytes by TUNEL assay

Cardiomyocytes seeded at a density of 2×10^4^ cells/well were treated either with culture medium (mock-treatment), 500 nM of AMD3100, 100 nM RANTES, 100 nM of Z-IETD-FMK, a preferential caspase 8 inhibitor-casp8i (Promega, USA), 100 nM Z-LEHD-FMK, a preferential caspase 9 inhibitor-casp9i (Promega, USA) for 1 hour at 37°C in the CO_2_ incubator. For UCLA1 inhibition, 100 nM of the aptamer was incubated with HIV-1 for 1 hour prior to infection. Cells were then exposed to HIV-1 subtype C clinical isolates at 30 ng/ml p24. After 3 days of incubation, the cell monolayers cultured in flat-bottom 96-well plates (Corning, USA) were fixed in 1% (w/v) paraformaldehyde and washed twice with 200 µl of a proprietary wash buffer (Guava Technologies, USA). The cells were then resuspended in 25 µl of a Brd-UTP labelling mix (Guava Technologies, USA) and incubated for 60 min at 37°C and stained with anti-BrdU-TRITC according to the manufacturer's instruction (Guava Technologies, USA). Data was analysed using the TUNEL assay program in the Cytosoft data acquisition and analysis software, Version 5.2 (Guava Technologies, USA). A total of 2000 events were acquired at a time and each well was acquired in triplicate. The results were displayed in a dot plot relating forward scatter (FSC) over fluorescence intensity (PM1) as well as a histogram (PM1 fluorescence intensity vs. number of events). The general apoptosis inducer staurosporin (Sigma-Aldrich, USA) was used as a positive control for terminal deoxynucleotidyl transferase dUTP nick end label (TUNEL) staining.

### Indirect immuno-fluorescence for Cytochrome c release detection

Cardiomyocytes were either mock-infected or infected with the HIV-1_CM9_ dual tropic strain for 2 days in the presence of either 100 nM of casp8i; 100 nM of casp9i or 100 nM of UCLA1. For all casp8i and casp9i, the inhibitors were added to the cell monolayer 2 hours prior to infection. The UCLA1 aptamer was first incubated with the virus for 1 hour before infection. Following 2 days of incubation the cells were harvested, fixed, stored and washed as above. A total of 2×10^4^ cells/well in 96-well, U-bottom plates, were treated with 5 µg/ml of mouse-anti human cytochrome *c* monoclonal antibody (Santa Cruz Biotechnology, Inc., USA) in 20 µl for 1 h at 37°C. The cells were then harvested by centrifugation, washed twice in an equal volume of PBS and counter stained with 1 µg/ml of Alexa Fluor 514 goat anti-mouse polyclonal antibody (Invitrogen, USA) for 30 min at room temperature. At the end of the incubation, rinsing buffer was added up to 200 µl and the cells were analyzed in the Guava EasyCyte Plus flow cytometer (Guava Technologies, USA).

### Caspase 8 and 9 enzyme activity and cell viability

Cardiomyocytes were seeded in 96-well tissue culture plates as previously described [21]. The cells were then exposed to eleven different conditions, namely 1 – media alone, 2 – conditioned media, 3 – HIV-1, 4 –100 µM of preferential caspase 8 inhibitor Z-IETD-FMK (casp8i) (Promega, USA), 5 –100 µM of preferential caspase 9 inhibitor Z-LEHD-FMK (casp9i) (Promega, USA), 6 – conditioned media and 100 µM Z-LEHD-FMK, 7 – conditioned media and 100 µM Z-IETD-FMK, 8 – conditioned media and 100 nM UCLA1 aptamer, 9 – HIV pre-incubated for 1 h with 100 nM of UCLA1 aptamer, 10 – HIV and 100 µM Z-LEHD-FMK, 11 – HIV-1 and 100 µM Z-IETD-FMK. The cells were harvested daily for 7 days followed by quantification of the caspases and cellular ATP levels, using the Caspase-Glo8, Caspase-Glo9 and the CellTiter-Glo luminescence-based detection kits as instructed by the supplier (Promega, USA). Wells containing media alone were used as controls for background luminescence and subtracted from the test values. The results were expressed as relative light units (RLUs) and plotted in a graph relating RLU to number of days.

### Cardiomyocytes apoptosis in HIV-infected macrophage co-cultures

Day 7 monocyte derived macrophages (MDM) seeded at a density of 2×10^4^ cells/well in a 24 well tissue culture plate (Corning, USA) were infected with R5 and dual-tropic viruses (30 ng/ml, p24) for 24 h in 200 µl of X-VIVO 10 medium (BioWhittaker) supplemented with 2% autologous serum. The cells were then washed 3 times with an equal volume of PBS followed by the addition of 2×10^4^ uninfected cardiomyocytes. To investigate apoptotic modulators, cells were cultured in the presence or absence of a TNF inhibitor (TNF-Ra, R&D Systems, USA), AMD3100, casp8i and casp9i. UCLA1 was pre-incubated with virus for 1 hour prior to infection. Controls included mock-infected cardiomyocyte-macrophage co-cultures. Following two days of exposure the cell monolayer was washed twice with warm PBS prior to trypsin (Lonza, USA) treatment. The strong adhesion properties of MDM were exploited to recover the less adherent cardiomyocytes. A 2 min trypsin treatment at 37°C was enough to recover over 95% of the cardiomyocytes but less than 3% of the macrophages, thereby minimizing cross-contamination. Purity of cardiomyocytes cultures and contamination by MDM was determined by flow cytometry using a 1∶200 dilution of a FITC-conjugated antibody directed against the CD4 cell surface receptor, which is absent on cardiomyocytes. Cardiomyocytes were then stained for TUNEL detection as described above.

## Supporting Information

File S1 **Infection of CM and PBMC with a panel of HIV-1 clinical isolates.**
(DOCX)
