# Supplementary material for: HIV-1 Subtype C Unproductively Infects Human Cardiomyocytes In Vitro and Induces Apoptosis Mitigated by an Anti-Gp120 Aptamer
Source: PLoS One. 2014 Oct 17;9(10):e110930. doi: 10.1371/journal.pone.0110930 (PMC4201581; doi:10.1371/journal.pone.0110930)
Supplement: File S1 — Infection of CM and PBMC with a panel of HIV-1 clinical isolates. (DOCX) [file pone.0110930.s001.docx]

**File S1: Infection of CM and PBMC with a panel of HIV-1 clinical isolates.**

**Experimental design**

The human cord-blood stem cell-derived cardiomyocytes (CM) and peripheral-blood mononuclear cells (PBMC) were isolated and cultured as detailed in the manuscript. CM and PBMCs seeded in 96-well plates (Celprogen and Corning, USA) at a density of 5×10^4^ cells/well were infected with a panel of HIV-1 clinical isolates (SW2, SW4, SW12, TM46b, CM9, DU179, RP1 and H9) at 200 ng/ml p24 concentrations. The infection was facilitated by spinoculation at 1220 g, 32 ºC for 1 h. The virus-infected cells were further incubated at 37 ºC, 5 % CO_2_ and harvested at 0, 2, 4, 24 and 72 h respectively. Supernatant harvested from HIV-exposed CM and PBMC was incubated with TZM-bl cells seeded at a density of 4000 cells/well in 96-well plates for 48 hours, 37°C, 5% CO_2_. Viral infection was determined with the addition of Bright-Glo luciferase substrate (Promega, Madison, Wisconsin, USA) to the samples in a 1:1 volume ratio and incubated for 2 minutes at room temperature to allow for complete cell lysis. The samples were transferred to a black, flat NUNC plate (Nunc^TM^, Thermo Fisher Scientific, Inc, MA, USA) and luminescence was detected at a 1000 ms integration time on a luminometer (Tecan i-control, 1.5.14.0, infinite F500, Männedorf, Switzerland). The luciferase enzyme expressed by infected cells interacted with the substrate to produce luminescence corresponded directly to the amount of viral infection. The results were analysed using Graphpad Prism (version 5.02).

**Results and discussion**

The data illustrates that although HIV-1 is capable of infecting both CM and PBMCs, the infection is weak and unproductive in CM (Figure S1A) compared to PBMCs (Figure S1B). Furthermore the data is in agreement with our initial findings that although HIV-1 can infect CM, it is incapable of completing its life cycle.

**Figure S1**. Infection of (A) CM and (B) PBMC with a panel of HIV-1 clinical isolates. Supernatant harvested from infected CM and PBMC at 0, 2, 4, 24 and 72 h was pre-incubated with TZM-bl cells for 48 hours, 37°C, 5% CO2. Results were expressed as relative light units (RLUs) and plotted relating RLU to hours post infection. Wells containing media alone were used as controls for background luminescence and subtracted from the test values.
